# Predictive Analysis of Yi-Gai-San's Multifaceted Mechanisms for Tremor-dominant Parkinson's Disease *via* Network Pharmacology and Molecular Docking Validation

**DOI:** 10.2174/0109298673291838240311075415

**Published:** 2024-06-14

**Authors:** Chih-Ting Lin, Lung-Yuan Wu, Fan-Shiu Tsai

**Affiliations:** 1 The School of Chinese Medicine for Post-Baccalaureate, I-Shou University, No. 8, Yida Rd., Jiaosu Village Yanchao District, Kaohsiung City, 82445, Taiwan;; 2 Department of Chinese Medicine, E-Da Cancer Hospital, No. 21, Yida Rd., Jiaosu Village Yanchao District, Kaohsiung City, 82445, Taiwan;; 3 Graduate Institute of Chinese Pharmaceutical Sciences, College of Chinese Medicine, China Medical University, No. 91, Hsueh-Shih Road, Taichung 40421, Taiwan;; 4 Wu Lung-Yuan Chinese Medicine Clinic, 3 F, No. 131, Section 1, Roosevelt Rd., Zhongzheng District, Taipei City, 10093, Taiwan

**Keywords:** Yi-Gan-San, Parkinson's disease, tremor-dominant, network pharmacology, molecular docking, *Uncaria rhynchophylla*

## Abstract

**Introduction:**

Based on comprehensive network-pharmacology and molecular docking analysis, this study was intended to unveil the multiple mechanisms of Yi- Gai-San (YGS) in treating the tremor-dominant subtype of Parkinson's disease (PD-DT). The compounds of YGS were meticulously analyzed, selected, and standardized with references to their pharmacological attributes. Its components included Gouteng (*Uncaria rhynchophylla*), Chaihu (*Radix Bupleuri*), Chuanxiong (*Chuanxiong Rhizoma*), Danggui (*Angelicae sinensis radix*), Fuling (*Wolfiporia extensa*), Baizhu (*Atractylodis macrocephalae rhizoma*), and Gancao (*Licorice, Glycyrrhizae radix*).

**Methods:**

We identified 75 active compounds within YGS. From these, we predicted 110 gene targets, which exhibited a direct association with PD-DT. PPI network results highlighted core target proteins, including TP53, SLC6A3, GAPDH, MAOB, AKT, BAX, IL6, BCL2, PKA, and CASP3. These proteins potentially alleviate PD-DT by targeting inflammation, modulating neuronal cell apoptosis, and regulating the dopamine system. Furthermore, GO and KEGG enrichment analyses emphasized that YGS might influence various mechanisms, such as the apoptotic process, mitochondrial autophagy, Age-Rage signaling, and dopaminergic and serotonergic synapses. The core proteins from the PPI analysis were selected for the docking experiment.

**Results:**

The docking results demonstrated that the most stable ligand-receptor conformations were kaempferol with CASP3 (-9.5 kcal/mol), stigmasterol with SLC6A3 (-10.5 kcal/mol), shinpterocarpin with BCL2L1 (-9.6 kcal/mol), hirsutine with MAOB (-9.7 kcal/mol), hederagenin with PRKACA (-9.8 kcal/mol), and yatein with GAPDH (-9.8 kcal/mol). These results provide us with research objectives for future endeavors in extracting single compounds for drug manufacturing or in-depth studies on drug mechanisms.

**Conclusion:**

From these computational findings, we suggested that YGS might mitigate PD-DT *via* “multi-compounds, multi-targets, and multi-pathways.”

## 1 INTRODUCTION

Parkinson's disease (PD) is characterized by a progressive neurodegenerative condition that leads to a range of debilitating motor symptoms, including resting tremor, bradykinesia, rigidity, and gait and postural abnormalities. In addition to these symptoms, there is often a concurrent presentation of non-motor manifestations, including gastrointestinal dysfunction (especially constipation), sleep irregularities, affective disorders (especially depression), cognitive decline leading to dementia, and generalized fatigue [1]. Among these, resting tremor, predominantly observable at rest, manifests as a slow, rhythmic tremor that typically initiates in one hand, foot, or leg and eventually affects both sides of the body. This is commonly recognized as a hallmark of PD and is referred to as the tremor-dominant subtype of Parkinson's disease (PD-DT), which clinicopathologically accounts for 8% of PD cases [2, 3]. The pathogenesis of PD-DT is associated with abnormalities in the cerebello-thalamo-cortical circuit. Studies have suggested that abnormal iron distribution within this circuit may lead to oxidative stress damage and neurotoxicity [4]. Furthermore, significant resting tremor has been linked to dopamine deficiency in the amygdala, along with the involvement of serotonergic raphe nuclei and noradrenergic locus coeruleus in the brainstem [5]. Additionally, 3-5% of cases can be attributed to monogenic inheritance associated with known Parkinson's disease-related genes, such as SNCA, LRRK2, VPS35, PRKN, PINK1, DJ-1, and GBA, while genetic variants collectively account for 16-36% of the non-monogenic heritable risk. Therefore, genomic research related to PD-DT is crucial for elucidating its therapeutic mechanisms [6, 7].

For most patients, treatment primarily focuses on symptomatic management, aiming to improve both motor and non-motor signs and symptoms. Mild PD-DT patients usually respond well to dopaminergic medications (*e.g.*, levodopa, dopamine agonists) for improvement of motor symptoms, while non-motor symptoms often require non-dopaminergic therapies (*e.g.*, MAO-B inhibitors, cholinesterase inhibitors, amantadine, selective serotonin reuptake inhibitors, and anticholinergics) [8]. However, as PD progresses to the later stages, patients often experience a significant reduction in the duration of medication effectiveness and encounter unpredictable motor fluctuations, commonly referred to as “on-off” phenomena [9]. Dyskinesia and various other side effects can arise from prolonged use of medications based on levodopa [10]. In such cases, surgical interventions or supportive therapies, such as traditional Chinese medicine (TCM) and non-pharmacological treatments (such as exercise, physical therapy, and speech therapy), may become more necessary. Extensive research on TCM has demonstrated its efficacy in treating or supporting the management of PD symptoms while exhibiting fewer and milder side effects [11-13].

Yi-Gan-San (YGS), also known as yokukansan, is a widely used traditional prescription in the treatment of neurodegenerative diseases. It consists of seven components, namely Chaihu (*Radix bupleuri*), Gancao (*Licorice, Glycyrrhizae radix*), Chuanxiong (*Chuanxiong rhizoma*), Danggui (*Angelicae sinensis radix*), Fuling (*Wolfiporia extensa*), Baizhu (*Atractylodis macrocephalae rhizoma*), and Gouteng (*Uncaria rhynchophylla*), combined in specific proportions of 5:5:8:10:10:10:10 [14]. YGS has been proven to reduce the frequency of insomnia-related awakenings and periodic limb movements in patients with Lewy body-related dementia and behavioral and psychological symptoms of dementia. This is highly biologically relevant to Parkinson's disease [15]. In addition, YGS has also been applied to address the involuntary, irregular choreoathetoid movements caused by dyskinesia [16]. Gouteng, the most crucial component in YGS, demonstrates remarkable neuroprotective effects by modulating IL-1β and brain-derived neurotrophic factor (BDNF), as well as the PI3K/Akt/mTOR signaling pathway, in experimental models of drug-induced PD [17, 18]. YGS significantly improved neuropsychiatric and anxiety symptoms in PD patients, including hallucinations, anxiety, apathy, mobility, and daily activities, as assessed by the Neuropsychiatric Inventory [19, 20]. In numerous randomized, multicenter, double-blind, placebo-controlled studies, YGS has consistently shown the absence of significant adverse effects, thus reinforcing its safety profile in clinical use [21]. Therefore, YGS may potentially provide an alternative treatment for PD-DT.

The results of network pharmacology analysis indicated that the potential mechanisms of YGS treatment involve the regulation of several neurotransmitter levels and the alleviation of neuroinflammation, which is consistent with its clinical application [22]. However, the mechanism against PD-DT has not been clarified due to the complex ingredients in YGS. Therefore, this study employed a systematic biology-based approach and molecular biology simulation technology to understand how YGS relieves PD-DT.

**Fig. (1):**
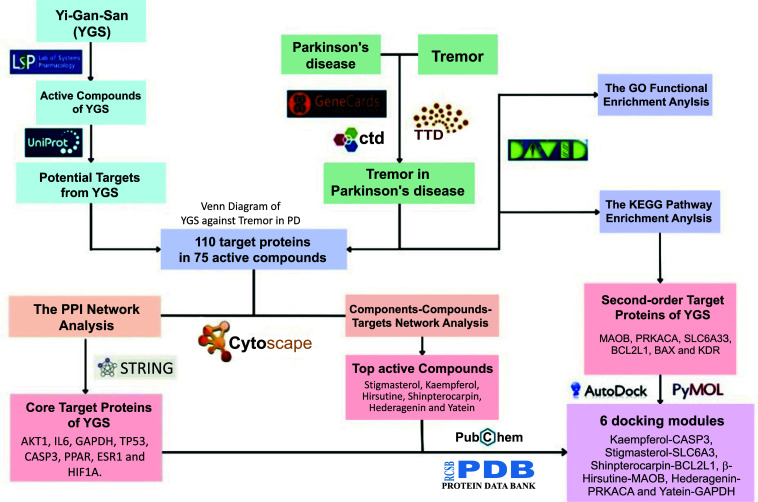
Flow diagram of work report.

To determine how YGS may protect against tremor in PD, several genetic, molecular and network pharmacology strategies were used. These include protein-protein interaction (PPI) analysis, Gene Ontology (GO), and Kyoto Encyclopedia of Genes and Genomes (KEGG) enrichment analysis. We also applied molecular docking technology to further illustrate the mode of action of YGS at a cellular level and how this could be adopted for future animal experiments (Fig. **1**).

## 2 MATERIALS AND METHODS

### 2.1 Screening for Active Compounds of YGS and Prediction of Compound-related Target Genes

The seven components of YGS (chaihu, gancao, chuanxiong, danggui, fuling, baizhu, and gouteng) were individually input into the Traditional Chinese Medicine System Pharmacology Database 2.3 (TCMSP database 2.3, https://tcmsp-e.com/tcmsp.php). The active chemical compounds of these components were screened by conditions of oral bioavailability value ≥ 30%, drug-likeness value ≥ 0.18, and drug half-life value ≥ 4 h. Within these criteria, “OB” and “HL” were frequently employed terms, while the “DL” value was calculated to optimize alignment with the ADME performance standards [23, 24]. Additionally, we conducted a literature search on the pharmacokinetics of YGS-related compounds to aid in the selection of major compounds. The target genes of active compounds were collected through the TCMSP database and translated through the UniProt database (http://www.uniprot.org) to collate the gene symbol name. TCMSP is built based on the systems pharmacology approach for herbal medicines, encompassing Chinese herbs along with their ingredient compounds, target proteins, and associated diseases [25]. Furthermore, there are several other databases related to TCM, such as TCMdatabase@Taiwan (http://tcm.cmu.edu.tw), TCM-Mesh database, and HIT database. However, these databases may have limitations in data download, lack sufficient quantifiable data, or face accessibility issues. In contrast, TCMSP stands out due to its provision of more comprehensive data and user-friendly download resources.

### 2.2 Collection of PD-PT-related Potential Target Genes

GeneCards database (https://www.genecards.org/) was used to search genes related to PD-PT by distributing both keywords, *i.e.*, “Parkinson’s Disease” and “Tremor”. Therapeutic Target Database (TTD, http://db.idrblab.net/ttd/) and Comparative Toxicogenomic Database (CTD, http://ctdbase.org/) were used to supplement the targets of PD-PT. The target genes induced by active compounds in YGS were cross-aligned with PD-related and tremor-related genes to obtain the core target genes curing PD-DT regulated by YGS. A Venn diagram was then prepared to understand the distribution of target genes to have some insight into the activity of YGS.

### 2.3 Construction and Topological Analysis of the Components-compounds-targets (CCT) Network

To understand the integrally molecular mechanisms of YGS, the graph of the CCT network was constructed by Cytoscape 3.9.1 software (Cytoscape Team, USA) with topological parameters of nodes: betweenness centrality (BC) and degree centrality [26]. In the CCT network, DC ≥ 7 and BC ≥ 0.012 were used as the screening criteria to obtain the key compounds. However, we selected at least one compound from each component that has the highest DC and BC level as the key compound set.

### 2.4 Protein-protein Interaction (PPI) Network Construction of Targets of YGS for PD-PT

One hundred ten core target genes of YGS for PD-PT were input to STRING database 11.5 (https://string-db.org/) to conduct a PPI analysis [27]. In the PPI network, DC ≥ 11 and BC ≥ 0.04 were used as the screening criteria to obtain the key protein targets in the PPI network. However, we selected and considered the PPI data with a combined score greater than 0.4 to optimally demonstrate protein-protein interactions while balancing the accuracy and quantity of the PPI. We did not adopt a higher threshold with combined scores above 0.7 or 0.9. The combined score expresses confidence in association-related evidence types for protein interactions, such as gene fusions, phylogenetic co-occurrence, and co-expression [28].

### 2.5 Function Enrichment Analysis of Gene Ontology (GO) and Kyoto Encyclopedia of Genes and Genomes (KEGG) Pathway

The 110 key protein targets of YGS for PD-PT were submitted to The Database for Annotation, Visualization, and Integrated Discovery (DAVID) bioinformatics resources (https://david.ncifcrf.gov/) to analyze GO function enrichment and KEGG pathway enrichment. GO terms and KEGG pathways with a *p*-value less than 0.05 were considered statistically significant and valid [29]. Furthermore, in the KEGG pathway enrichment analysis, we contrasted the protein-target distribution in the biological pathways of our research between YGS and common PD clinical medications, such as dopamine receptor agonists, COMT inhibitors, MAO-B inhibitors, and amantadine.

### 2.6 Preparation for the Molecular Docking Analysis between YGS Compounds and PD-PT Protein Targets

Molecular docking was analyzed using AutoDock 4.2 software, which employs the Lamarckian genetic algorithm [30]. All the three-dimensional structures of target proteins were downloaded from the RCSB PDB database (https://www.rcsb.org/), including GAPDH (pdb id: 6YND), AKT1 (pdb id: 1UNQ), IL6 (pdb id: 7NXZ), CASP3 (pdb id: 4JJE), TP53 (pdb id: 6GGF), BCL2L1 (pdb id: 7LH7), BAX (pdb id: 5W60), PRKACA (pdb id: 7V0G), MAOB (pdb id: 6FW0), and SLC6A3 (pdb id: 6M0Z). In order to ensure the active conformation, we carefully selected target protein models from the RCSB PDB database obtained through X-ray crystallography techniques. Additionally, we considered only protein models with a resolution of less than 3Å to certify appropriate crystal quality [31]. For X-ray crystallography models, we employed an additional filtering criterion, requiring the value of R-free to be below 0.25, further validating the accuracy of the selected models [32]. These pre-docking preparation parameters are presented in Table **1**. Before performing the molecular docking experiments, we eliminated extraneous molecules from the chosen target protein models, including H_2_O, zinc ions, S-sulfinylation, inositol-1,3,4,5-tetrakisphosphate, L-norleucine, glycerol and antibody fragments. Simultaneously, we preserved the region corresponding to the orthostatic site of the original model and substituted it with our active compounds for the docking procedure. All docking simulations were run with default settings, generating a maximum of 9 different binding modes. We picked the docking result with the largest binding energy absolute value as the best receptor-ligand interaction simulation.

## 3 RESULTS

### 3.1 Potential Target Genes Analysis of PD-PT and YGS

For the potential YGS-related PD-DT target genes analysis, 480 genes for PD, 400 genes for tremor from the GeneCards database, TTD and CTD database, and 283 genes for YGS from the TCMSP database were observed. Based on the Venn analysis in Fig. (**2**), there were 227 overlapping potential target genes between PD and tremor. These 227 genes were further surveyed with YGS potential target genes. The comparison of the potential target genes is shown in Fig. (**2**). Results revealed 110 overlapping potential target genes between PD-DT and YGS (Table **1**). Subsequently, we retrospectively traced back these overlapped potential targets from the databases and identified 75 compounds in YGS capable of regulating these genes. Moreover, we cross-referenced existing reference literature containing HPLC data for each component in YGS to confirm the presence of these compounds. These compounds are referred to as active compounds. The active compounds in YGS and their corresponding potential target genes and pharmacokinetic information are summarized in Table **2**. The corresponding structural formula of each active compound is listed in Fig. (**3**).

### 3.2 Network Construction and Topological Analysis

To understand the correlations of YGS and its seven components with potential target genes against PD-PT, a correlation complex network was constructed. As shown in Fig. (**4**), there were 193 nodes (consisting of one disease, seven components in YGS from the TCMSP database, 75 active compounds and 110 potential target genes) and 775 edges. Results showed that the size of green squares could reflect the statistical significance of the compound terms. According to the topological analysis, active core compounds with more than the criterion of degree ≥ 7 and BC ≥ 0.012 were summarized. Also, at least one compound should be singled out in one component to express its importance, such as hederagenin (F4) from fuling and scopoletol (B7) from baizhu. Table **3** presents that the core active compounds were selected based on node topological parameters. Above all, the most important ten active compounds of YGS in distinct components are stigmasterol in chaihu/danggui, kaempferol in chaihu, isorhamnetin in chaihu, beta-sitosterol in danggui, hederagenin in fuling, shinpterocarpin in gancao, 4-hydroxy-3-butylphthalide in chuanxiong, yatein in gouteng, palmitic acid in fuling/danggui, scopoletol in baizhu, and hirsutine in gouteng.

### 3.3 Construction and Analysis of the PPI Network

We incorporated 110 target protein molecules and then conducted a PPI network analysis. In the PPI analysis, five target proteins, SLC25A13, SLC22A5, PCYT1A, NEK9, and GBA2, did not establish interactions with the comprehensive set of target proteins. Furthermore, the combined scores for these five proteins fell below our selection threshold. As a result, as shown in Fig. (**5**), the network is comprised of only 105 nodes (representing target proteins) and 1308 edges (indicating interactive relationships). The result revealed five distinct clusters, each represented by a unique color (pink, blue, green, purple, and yellow). These clusters demonstrated a significant functional association with neuron-related apoptosis responses, synaptic neurotransmitter modulation, Age-Rage signaling, and focal adhesion. These include the following: the dopaminergic/serotonergic synapse related cluster (including solute carrier family 6 member 3 (SLC6A3), D1A dopamine receptor (DRD1), Cytochrome P450 3A4 (CYP3A4) and amine oxidase B (MAOB), the apoptosis-related pathway cluster (including alpha serine/threonine-protein kinase (AKT1), Caspase-3 (CASP3), TP53-binding protein (TP53), Bcl-2- like protein 1 (BCL2L1), apoptosis regulator (BAX) and cAMP-dependent protein kinase catalytic subunit alpha (PRKACA), the Age-Rage signaling pathway related cluster (including interleukin-6 (IL6), C-X-C motif chemokine 8 (CXCL8) and (VEGFA), the focal adhesion-related cluster (including gamma-aminobutyric acid receptor subunit alpha 2 (CAV1), muscarinic acetylcholine receptor 1 (KDR) and (EGFA), and the biosynthesis metabolic pathway related cluster (including gamma-aminobutyric acid receptor subunit alpha 2 (GAPDH) and (PYCR1). After screening the network properties criterion of degree ≥11 and BC ≥ 0.04, 10 proteins were selected to be the core target genes in the PPI network analysis (Table **4**). Besides, from the width and transparency of the edges determined by the combined score, it was observed that not only do the target proteins within the same protein cluster exhibit interaction tendencies, but there are also notable interaction tendencies between different protein clusters. Such observations suggest that in the therapeutic mechanism of YGS, there might exist shared regulatory pathways. A single protein's regulation may not be limited to just one mechanism.

### 3.4 Functional and Pathway Enrichment Analysis

GO functional enrichment analysis is presented in Fig. (**6**) and Table **5**. The biological process annotations included inflammatory response, response to lipopolysaccharide, negative regulation of cell proliferation, apoptotic process, *etc.* IL6, in conjunction with lipopolysaccharide (LPS) and other inflammatory stimuli, can induce a pro-inflammatory response. TP53 and CASP3 are both involved in regulating apoptosis, with CASP3 being the central executor enzyme of apoptosis. Meanwhile, interactions between BAX and the Bcl-2 family can influence the apoptotic process. Furthermore, AKT1 plays a pivotal role in both positive and negative regulation of inflammatory responses and cell proliferation. The cellular component annotations showed enrichment in neuron projection, an integral component of the synaptic membrane, mitochondrial outer membrane, and focal adhesion. BAX is directly associated with the outer mitochondrial membrane, whereas SLC6A3 is linked to the synaptic membrane. The molecular function annotation revealed protein kinase binding, transcription factor binding, oxidoreductase activity, cytokine activity and protein phosphatase binding, among others. AKT1 and PRKACA both possess protein kinase binding functions. IL6 exhibits cytokine activity, while GAPDH and MAOB display oxidoreductase enzymatic activity. A total of 84 pathways were identified in the KEGG signal pathway enrichment analysis. The top 20 pathways were selected and are presented in Fig. (**7**) and Table **6**. We identified a series of pathways, categorically organized into three predominant functional groups. Within the realm of the inflammatory response, we noted the inclusion of pivotal pathways, such as the AGE-RAGE signaling cascade, HIF-1 signaling dynamics, PI3K-Akt signal transduction, NF-kappa B signaling, and the intricate processes of apoptosis. Furthermore, the neural function cluster distinctly highlighted pathways encompassing the dopaminergic synaptic pathway, serotonergic synaptic pathway, and tryptophan metabolic process, coupled with neurodegeneration-centric pathways. Conclusively, the ensemble encapsulating intercellular junction dynamics prominently featured the mechanisms of the focal adhesion and the gap junction pathway.

### 3.5 Potential Target Spot Configuration of YGS in Different Pathways

**Fig. (8):**
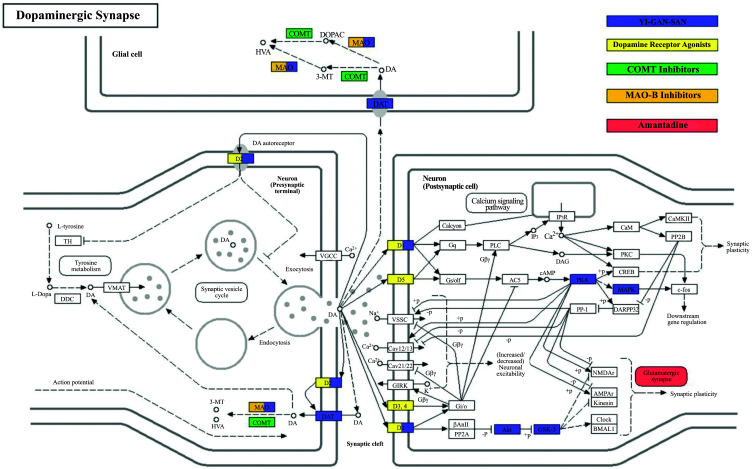
Potential target spots of YGS/dopamine receptor agonists / COMT inhibitors / MAO-B inhibitors/amantadine on regulating dopaminergic synapse. Arrows indicate activation effects, and T-arrows indicate inhibition effects. The spots in purple are targets regulated by YGS; the yellow spots indicate regulation by dopamine receptor agonists; the green spots denote regulation by COMT inhibitors; the orange spots signify regulation by MAO-B inhibitors, and the red spots represent regulation by amantadine.

The dopaminergic synapse signaling pathway is the most critical aspect of the potential mechanisms of YGS treatment. According to the findings from the KEGG analysis (Fig. **8**), YGS exhibits the capability to activate dopamine receptors, resembling the effects observed with conventional dopamine receptor agonists. Notably, YGS exhibits selectivity in its activation, specifically targeting the D1 and D2 dopamine receptors. YGS also demonstrates the ability to regulate MAO, thereby attenuating the catabolism of dopamine within synaptic clefts. This action results in an elevated concentration of dopamine in neuronal cells, mirroring the function observed in MAO-B inhibitors. The mechanisms of action of other prevalent PD medications differ notably from that of YGS. For instance, COMT inhibitors hinder the COMT enzyme from breaking down dopamine. Additionally, Amantadine, a type of glutamate antagonist, is utilized to promote dopamine release, thus enhancing dopamine activity in the brain and helping to reduce dyskinesia. As shown in Fig. (**8**), it is evident that within the dopaminergic pathway, YGS treatment has a broader protein regulation scope compared to other PD drugs. This indicates that YGS encompasses various essential compounds that modulate different proteins with the aim of rectifying PD-DT rather than relying on a singular component for its therapeutic mechanism. Furthermore, within the Age-Rage signaling and cell-apoptosis relevant pathways, YGS demonstrates modulation on a predominant set of target proteins and exerts influence over several sub- pathways, as depicted in Fig. (**9**). Collectively, these findings suggest that YGS may mitigate PD-DT *via* a mechanism characterized by its “multi-compounds, multi-targets, and multi-pathways” approach.

### 3.6 Molecular Docking Analysis of Compounds-targets Interaction

Ten selected compounds (kaempferol and stigmasterol in chaihu component, stigmasterol, palmitic acid and beta-sitosterol in danggui component, shinpterocarpin in licorice component, hirsutine and yatein in gouteng component, palmitic acid and hederagenin in fuling component, 4-hydroxy-3-butylphthalide in chuanxiong component, and scopoletol in baizhu component) were obtained after screening from the CCT network, as mentioned in Table **3**. These compound ligands were docked with GAPDH (Glyceraldehyde-3-Phosphate Dehydrogenase), AKT1 (RAC-alpha serine/threonine-protein kinase), IL6 (Interleukin 6), CASP3 (Caspase 3), TP53 (Tumor Protein p53), BCL2L1 (BCL2-like 1), BAX (BCL2-associated X protein), PRKACA (Protein kinase A catalytic subunit alpha), MAOB (Monoamine oxidase B), and SLC6A3 (Sodium-dependent dopamine transporter). Generally, the higher the binding energy absolute value, the greater the protein binding affinity of the docking pair. As illustrated in Fig. (**10**), the binding affinities of kaempferol to CASP3 (-9.5 kcal/mol), stigmasterol to SLC6A3 (-10.5 kcal/mol), shinpterocarpin to BCL2L1 (-9.6 kcal/mol), hirsutine to MAOB (-9.7 kcal/mol), hederagenin to PRKACA (-9.8 kcal/mol), and yatein to GAPDH (-9.8 kcal/mol) were notably lower than -9.5 kcal/mol, indicating greater stability in the conformation of protein binding for these key compound-target pairs. Additionally, these pairs exhibited a more prominent presence of visualized polar hydrogen bonds compared to other molecular docking pairs (at least one pair), further supporting their stable binding interactions. In the findings presented in Fig. (**11**), kaempferol established hydrogen bonds with CASP3 at residues HIS-121, SER-205, and ARG-207. Shinpterocarpin formed hydrogen bonds with BCL2L1, specifically at ARG-146, while Hederagenin engaged with PRKACA at SER-53 and THR-183. Additionally, hirsutine displayed interactions with MAOB residues TYR-393 and GLN-392. Hydrogen bonding of stigmasterol was evident with SLC6A3 at GLN-322, and yatein showed interactions with GAPDH at ALA-238 and ASN-287. These interactions emphasize the intricate molecular relationships between the active compounds and respective protein targets. These computer-simulation results of molecular docking confirmed the network pharmacology analysis on how YGS components influence PD-DT and laid the foundation for further study on its pharmacological mechanisms. Although the candidate pairs for our molecular docking analysis differ from the target-compound pairs shown in Table **2**, this molecular docking analysis will still be performed to validate the statistical results obtained from CCT and PPI network analyses. It will also facilitate the execution of novel target-compound experiments with divergent results from the extant databases.

## 4 DISCUSSION

**Fig. (11):**
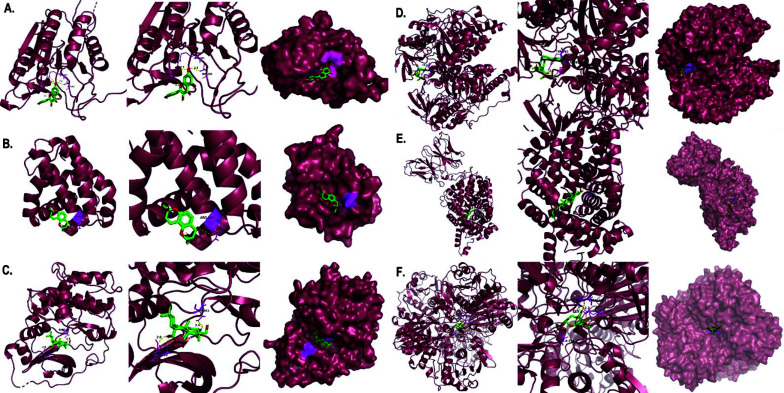
3D molecular docking visualization results of target proteins with active compounds of YGS. (**A**). kaempferol with CASP3 (4JJE); (**B**). shinpterocarpin with BCL2L1 (7LH7); (**C**). hederagenin with PRKACA (7V0G); (**D**). hirsutine with MAOB (6FW0); (**E**). stigmasterol with SLC6A3 (6M0Z); (**F**). yatein with GAPDH (6YND). Active compounds are represented by green-red stick models, and the secondary structure of the protein is represented by a dark burgundy color ribbon. The yellow dotted lines between active compounds and target proteins represent the polar hydrogen bond bonding sides. In addition, surface conservation analysis emphasized how receptor compounds bind to ligand proteins.

In our study utilizing the TCSMP and TCMID database, we identified a total of 75 compounds contained in YGS that are implicated in the treatment of PD-TD. These compounds exhibit reasonably favorable pharmacokinetic absorption rates and blood concentration profiles. However, the plasma maximum concentration of YGS demonstrates a dose-dependent increase ranging from 0.261 to 1.980 ng/mL, with a nearly similar time to reach peak concentration (tmax = 0.42-0.67 hours). The area under the plasma concentration-time curve also exhibits a dose-dependent increase within the range of 0.441-6.79 ng h/mL. The half-life of different components of YGS is approximately between 1.4 and 9.11 hours, with alkaloid compounds (such as isocorynoxeine and rhynchophylline) showing higher HL after YGS intake [33]. Subsequent CCT network analysis highlighted that 10 of these compounds (Table **3**) hold statistical significance and play crucial roles in the pharmacological mechanism of YGS. Stigmasterol, a lipophilic plant-derived sterol, has been shown to modulate neuronal responses to post-hypoxia and oxidative insults. Its protective mechanisms include enhancing AKT phosphorylation, reducing hyper-activation of the cdk5/p25 signaling pathway, and initiating cellular mitochondrial autophagy [34, 35]. These mechanisms present pivotal counteractions to the mitochondrial dysfunctions and neuroinflammatory damages associated with Parkinson's disease, corroborating seamlessly with our results from the GO functional enrichment analysis. Despite the solubility challenges posed by stigmasterol, its therapeutic potential has been notably amplified by conjugating it with soy polysaccharides to overcome such limitations [36]. Kaempferol exhibits significant therapeutic potential for Parkinson's disease (PD), showing mechanistic similarities with stigmasterol. Specifically, kaempferol actively modulates monoamine levels within the striatum and substantia nigra, reduces the levels of pro-inflammatory cytokines, and demonstrates antioxidative abilities [37]. Notably, its modulation of caspase cleavage augments mitochondrial autophagy, which aligns with our molecular docking analysis result (Fig. **11A**) [38, 39]. This positions kaempferol as a crucial autophagy enhancer in the therapeutic context of PD. Additionally, the extracted compound hederagenin exhibited neuroprotective effects in a Parkinson's disease mouse model. Through the MAPK-mTOR pathway-dependent autophagy, it reduces levels of mutated huntingtin protein and toxic fibrillar aggregates of α-synuclein [40]. Furthermore, shinpterocarpin, one of the compounds in *Glycyrrhizae radix*, shows potential in inhibiting the hyperphosphorylation of the MEK-ERK1/2 pathway and alleviating mitochondrial oxidative stress. Consequently, it reduces neuronal cellular apoptosis, highlighting its potential therapeutic role in Parkinson's disease [41]. However, limited neurobiological evidence suggests that yatein, a lignan from *Uncaria rhynchophylla* ethanol extract, may protect hippocampal neurons in albino rats from D-galactose-induced damage, possibly through its antioxidant properties that reduce lipofuscin accumulation [42]. Palmitic acid, obtained through the ethyl acetate fraction, has shown the ability to rejuvenate dopaminergic neuron functionality, enhance dopamine-dependent behaviors, and alleviate oxidative stress. Moreover, it has demonstrated proficiency in reducing the aggregation of α-synuclein, highlighting its potential therapeutic significance in nutritional strategies for PD [43]. Additionally, palmitic acid and scopoletol inhibit the proliferation of cancer cells by targeting the PI3K/AKT signaling pathway. While this assertion is consistent with our GO and KEGG enrichment analysis, there is currently no substantial evidence to suggest a direct correlation or support for this mechanism in the treatment of PD [44, 45]. Hirsutine, derived from *Uncaria rhynchophylla*, reduces the production of pro-inflammatory cytokines, such as IL-6, TNF-α, and IFN-γ in LPS-mediated neuroinflammatory models and mitigates LPS-associated hippocampal cell death [18, 46]. Furthermore, hirsutine modulates levels of dopamine and its associated metabolites, increases the BCL2/BAD ratio, and enhances the phosphorylation of AKT, mTOR, and MAPK signaling proteins [47]. Such mechanisms highlight the potential protective role of the aforementioned compounds for dopaminergic neurons in Parkinson's disease. However, compared to the aforementioned compounds, there is no literature on beta-sitosterol and 4-hydroxy-3-butylphthalide, which validates our results. This suggests that these compounds may be the focal points of our forthcoming intensive and deeper studies.

The results from both the GO and KEGG enrichment analyses, as well as the mechanisms of compounds revealed in the CCT network analysis, suggest that the treatment of PD-DT is associated with combating neuroinflammation, cellular apoptosis, and mitochondrial health. Likewise, pivotal proteins identified in the PPI network analysis, including AKT, CASP3, BCL2, BAX, GAPDH, TP53, and IL6, are involved in the regulatory processes of these mechanisms. For instance, YGS enhances the binding affinity between nerve growth factor and the TrkA receptor, subsequently activating the phosphorylation of AKT and ERK1/2, promoting neurite outgrowth activity [48]. YGS and corynoxine (G5) notably reduce the activity of caspase-3 and protect dopaminergic neurons in PD animal models from the toxicity of MPTP [49, 50]. The oxindole alkaloids derived from *Uncaria rhynchophylla* in YGS significantly inhibit caspase-3 activation induced by H_2_O_2_, suppress the upregulation of BAX, and promote the downregulation of BCL2. This leads to an enhanced BCL2/BAX ratio, increased SOD activity, and reduced MDA levels, stabilizing the mitochondrial membrane and decreasing DNA fragmentation [51, 52]. Kaempferol distinctly suppresses the activity of caspase-3 and caspase-9, and this inhibitory effect exhibits a dose-dependent relationship, similar to the effects of donepezil hydrochloride [53]. Research findings suggest that, compared to the control group, there was a marked increase in the nuclear localization of GAPDH and Tp53 within the substantia nigra region of PD brains [54]. These apoptotic signaling markers further induced the activation of caspase 3 and the elevation of BAX levels [55]. Consequently, when YGS effectively reduces the nuclear translocation of these apoptotic signaling markers, it may help mitigate the progression of neurodegenerative changes. This corroborates the results from our PPI network analysis. Regarding the promotion of anti-inflammatory properties in neuronal cells, YGS actively inhibits the inflammatory amplification mechanism inherent in the p38/MAPK/NF-κB signaling pathway. It reduces the levels of pro-inflammatory products, such as IL-6, TNFα, and IL-8, while also downregulating the expression of COX-2, thereby effectively curtailing the expansion of the inflammatory response [56]. For instance, the extract from Atractylodis rhizoma displays a pronounced ability to suppress IL-6 [57]. Importantly, patients with PD-DT exhibited significantly elevated levels of IL-6 in both cerebrospinal fluid and serum compared to patients characterized by postural instability/gait difficulty. This elevation coincides with increased iron levels in the CSF and L-ferritin levels in serum, underscoring the potential interplay between abnormal iron deposition in the brain and inflammation as a mechanism for Parkinsonian tremor [58]. In the future, serum levels of IL-6 and L-ferritin may serve as diagnostic markers to evaluate the appropriateness of YGS administration in PD-DT patients. Our KEGG enrichment analysis indicates that within the therapeutic mechanism of YGS for PD-DT, the AGE-RAGE signaling regulation plays a pivotal role in inhibiting chronic neuronal inflammation. Additionally, concentrations of RAGE ligands showed significant elevations in the substantia nigra and cerebrospinal fluid of PD patients. Remarkably, when blocking the AGE-RAGE signaling in animal models through RAGE gene knockout or the use of RAGE inhibitors, substantial protection of dopaminergic neurons from PD-like symptoms was observed [59]. A standout component within YGS, *Uncaria rhynchophylla*, demonstrates the ability to downregulate RAGE and p-JAK2, concurrently inhibiting the concentrations of AGEs, MDA, and ROS [60].

In the therapeutic appliance of PD, strategies primarily pivot around augmenting or emulating dopamine functionality, mitigating its metabolic degradation, and influencing associated neurotransmitters to alleviate clinical manifestations. The overarching objective is to address the diminished dopamine concentration inherent in the nigrostriatal circuitry. Pertinent therapeutic agents encompass Levodopa (L-DOPA), dopamine receptor agonists, MAO-B inhibitors, COMT inhibitors, NMDA receptor antagonists, and anticholinergic medications. Our KEGG enrichment analysis results align with the notion that YGS exerts a pivotal modulatory effect on proteins in dopaminergic synapses, including MOAB, DAT, DRD2, and DRD1. Molecular docking findings showed that MOAB-hirsutine and DAT-stigmasterol possess notable molecular affinities. This suggests that hirsutine might share pharmacological similarities with MAO-B inhibitors. For instance, when comparing hirsutine with safinamide, a kind of MAO-B inhibitor, the drug-likeness parameters of hirsutine, such as mol weight (368.47/302.34), number of hydrogen bond acceptors (32/23), topological polar surface area (54.56/64.35), and octanol/water partition coefficient (logP = 3.75/3.46), are remarkably congruent [61, 62]. Concurrently, stigmasterol emerges as a potential candidate as a DAT potentiator, which could serve as a promising experimental target for future drug trials. The synergistic effects with L-DOPA suggest that YGS may play an adjunctive role in PD therapy, potentially enhancing the therapeutic efficacy of existing medications or attenuating their associated side effects. Moreover, studies have indicated that compounds extracted from *Uncaria rhynchophylla* effectively inhibit MAO-B activity. The suppression of MAO-B activity can enhance dopamine levels in the substantia nigra, offer neuroprotective benefits, ameliorate non-motor-related symptoms, such as emotional and cognitive dysfunctions, and synergize with L-DOPA to prolong the duration of dopamine action [63]. Another study revealed that YGS significantly amplifies and extends the L-DOPA-induced motor and axial abnormal involuntary movements. It also augments dopamine production in a manner analogous to the COMT inhibitor entacapone, with the primary COMT inhibitory activity attributed to the alkaloids, corynoxeine and geissoschizinc acid found in *Uncaria rhynchophylla* [64]. Although our study did not highlight the COMT inhibitory effects of YGS, this might be attributed to the potential omission of compounds that regulate this protein. Various indole alkaloids identified in *Uncaria rhynchophylla* have been found to exhibit significant dopamine D2 receptor antagonistic activity. Such effects can aid in balancing dopamine activity, mitigating or eliminating non-motor symptoms like hallucinations and delusions that arise due to hyperactivity induced by drugs, such as L-DOPA [65]. Beyond the regulation mechanisms of dopaminergic synapses, the severity of resting tremor in PD patients is closely associated with the deficiency of serotonin transporters in the raphe nuclei rather than with dopamine loss in the striatum [66]. This also accounts for the disappearance of tremors during sleep, co-occurring gastrointestinal symptoms, such as constipation, and the subpar response to L-DOPA medication [67]. Furthermore, serotonin levels in the blood of PD-TD patients are lower than those with non-tremor dominant PD, and the availability of serotonin transporters is reduced among PD patients, with the severity of the tremor correlating with its depletion [66]. Some studies advocate exploring drugs that modulate the serotonin system, such as clozapine, for the treatment of Parkinson’s tremors [68]. However, our PPI network and KEGG enrichment analyses validate that YGS possesses the capacity to regulate serotonin transporter and serotonergic synaptic pathways, suggesting that it may offer therapeutic selectivity for pronounced tremor symptoms in PD patients.

Our study, employing network pharmacology and molecular docking analysis, presents several limitations. Unfortunately, in the existing traditional Chinese medicine database, there is no indication of the relevant components of YGS being able to regulate target proteins highly correlated with PD, such as LRRK2, SNCA, PRKN, PARK7, GBA, PINK1, *etc.*, which are the latest research findings. This may be attributed to the fact that molecular experiments related to YGS and PD have not been widely conducted, thereby preventing effective linkage of the pharmacological mechanisms of YGS with the latest target proteins in network pharmacology. Yet, our network pharmacology approach struggles to quantify this relationship in terms of dosage. This underscores the need for further advancements, particularly in the areas of novel algorithmic developments, meticulously designed animal studies, and clinical applications. Such endeavors will enrich our comprehension of disease and syndrome regulatory mechanisms and shed light on the biological underpinnings of YGS [69, 70]. To encapsulate, our findings are primarily computational and derive from clinical empirical observations without the backing of rigorous animal or clinical trial validations. Additionally, the search results obtained by our systems biology experiment are based on data up to 2022. Our subsequent experimental design aims to ascertain the neuroprotective effects of YGS, or its extracted compounds, on dopaminergic neurons in PD-induced animal models. One particularly important step is to validate the predicted protein-protein interaction analysis results. To achieve this, we will explore techniques, such as co-immunoprecipitation (Co-IP), surface plasmon resonance (SPR), or fluorescence resonance energy transfer (FRET), to gather further evidence. Additionally, we intend to evaluate neuroprotection by assessing dopamine or dopamine metabolic product levels in serum or substantia nigra tissue, as well as evaluating various inflammatory markers and cellular apoptosis pathway factors, including AKT1, IL6, CASP3, TP53, BCL2, and BAX. Concurrently, a comparative analysis will be conducted to study the pharmacological properties of existing dopamine-affecting drugs in comparison to YGS. We will further explore the regulatory mechanisms of YGS on DRD2, DAT, MOAB, or COMT and then extend the discussion to determine whether YGS genuinely modulates serotonergic function.

## CONCLUSION

In this study, we utilized network pharmacology and molecular docking strategies to identify the potential compounds and protein targets of YGS in PD-DT treatment. Our findings indicate that the therapeutic mechanisms underlying YGS encompass the inhibition of nerve inflammation, the execution of mitochondrial neuroprotective effects, the prevention of neuronal apoptosis, and the modulation of the dopaminergic transmission system and their interactions. Key active compounds, namely kaempferol, stigmasterol, palmitic acid, beta-sitosterol, shinpterocarpin, hederagenin, 4-hydroxy-3-butylphthalide, hirsutine, scopoletol, and yatein, were identified. These compounds then targeted significant proteins, including TP53, SLC6A3, GAPDH, MAOB, AKT, BAX, IL6, BCL2, PKA, and CASP3, as revealed in the PPI and CCT networks. Collectively, our results demonstrated that YGS exerts its therapeutic effects *via* a comprehensive “multi-compounds, multi-targets, and multi-pathways” mechanism. Future research must build on these findings. The computational and system analyses presented herein offer a solid theoretical framework for subsequent studies.

## AUTHORS' CONTRIBUTIONS

L.Y.W. contributed to the conceptualization of the study. C.T.L. performed the formal analysis and contributed to the writing of original draft. F.S.T. and L.Y.W. were involved in the project administration. F.S.T. contributed to the writing, reviewing, and editing. C.T.L. prepared the figures and tables.

All authors have read and agreed to the published version of the manuscript.

## Figures and Tables

**Fig. (2):**
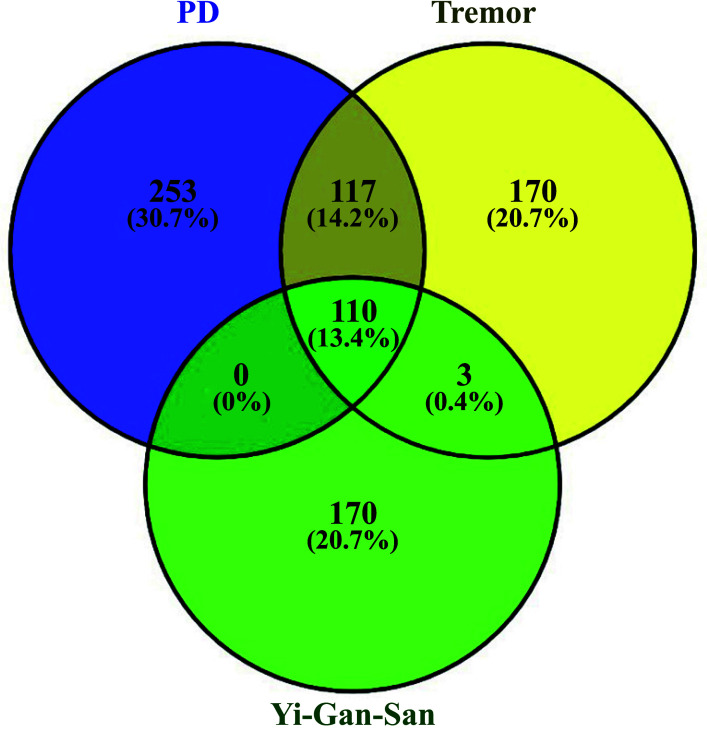
Venn screening of potential target genes between YGS (Yi-Gan-San) from TCMSP database (entering seven components separately) and PD-DT.

**Fig. (3):**
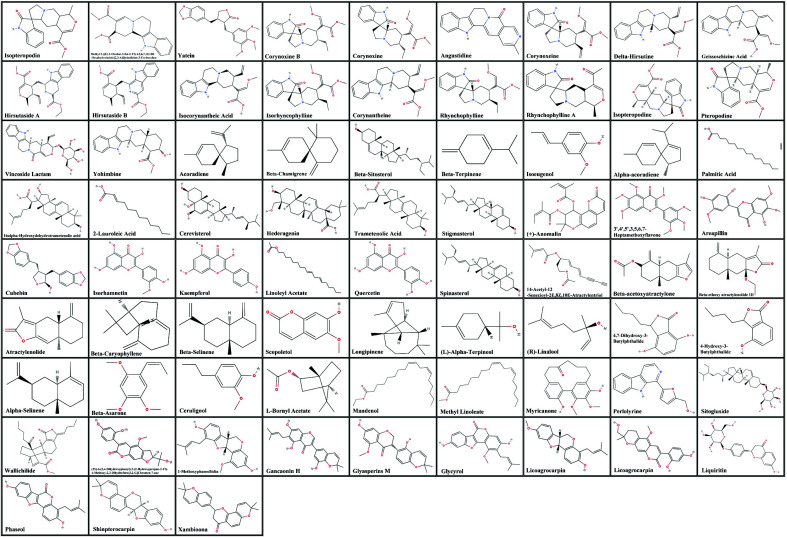
The corresponding chemical structures of active compounds.

**Fig. (4):**
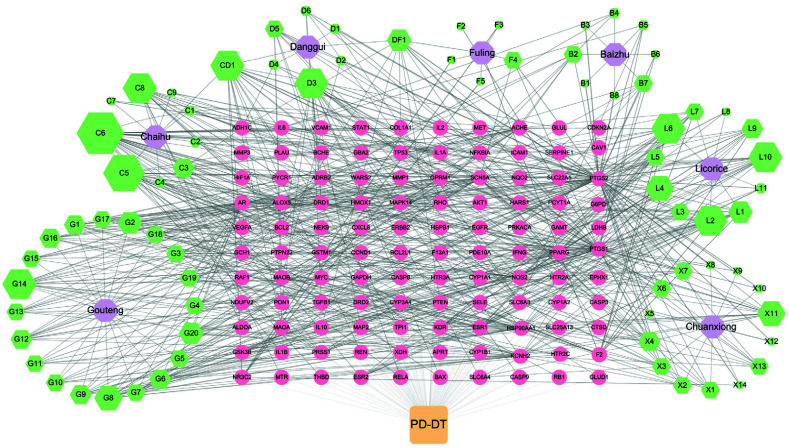
Components-compounds-targets networks of YGS and PD-DT. The orange square is the disease, PD-DT. Purple hexagons are seven components of YGS (from the TCMSP database). Green hexagons indicated the 75 active compounds of the YGS components. Yellow circles indicate the 110 potential target genes. The size of nodes represents only the DC of active compounds (Other objects were just displayed in a certain size).

**Fig. (5):**
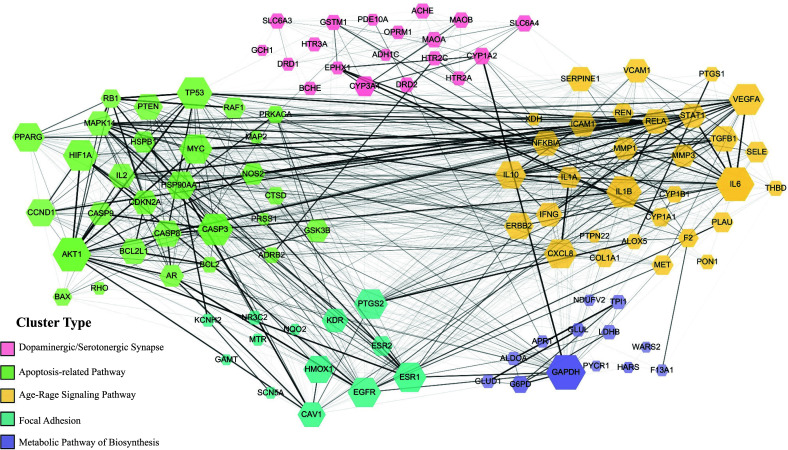
The interactive PPI network among target proteins of YGS against PD-DT. There was a positive proportional relationship between the node size and the node degree criterion. The diverse width and transparency of the edges, which are determined by the combined score of two target proteins, represent the interaction extent of each node. Moreover, proteins with similar bio-functionality were classified into five distinct clusters.

**Fig. (6):**
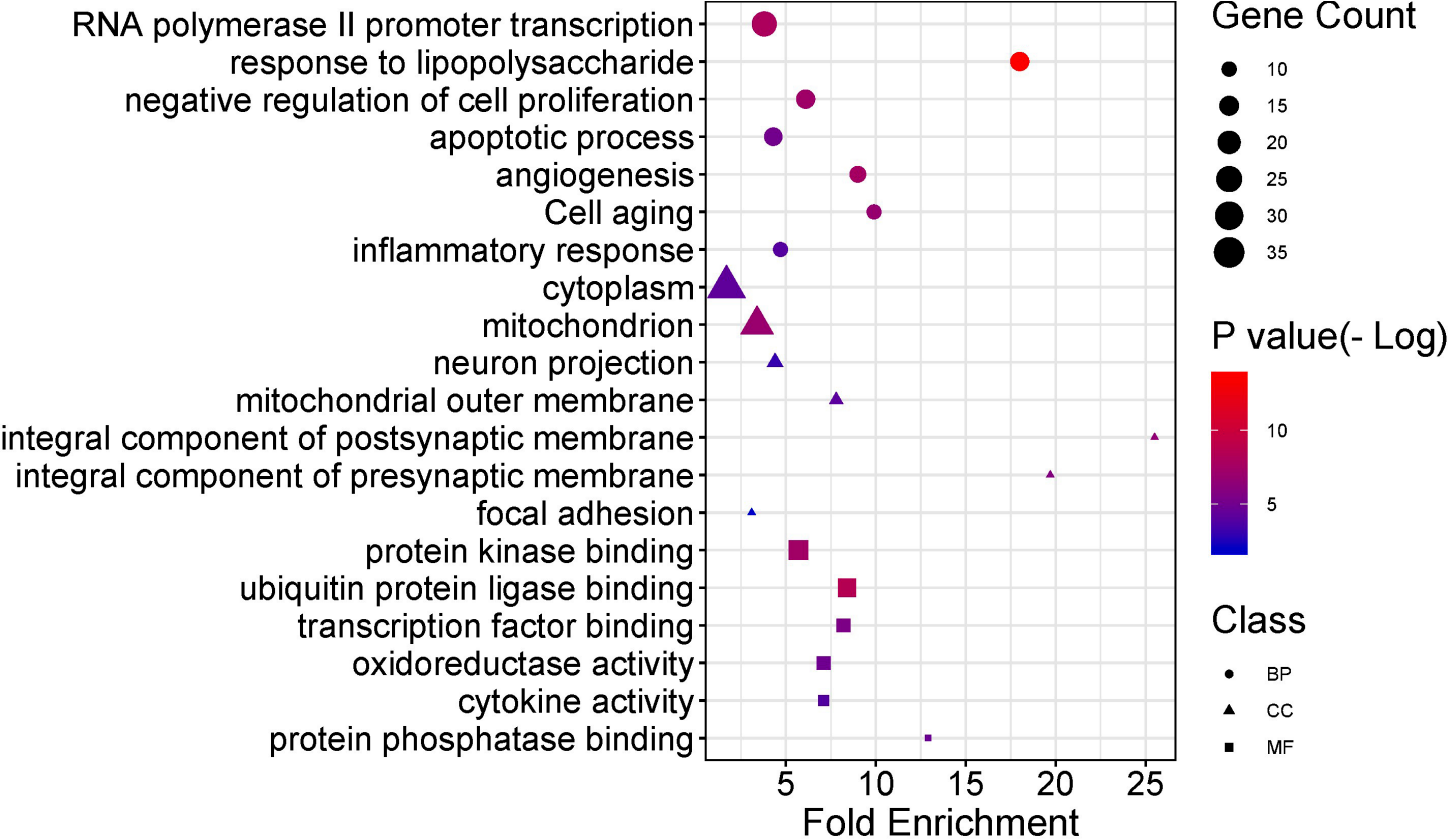
Functional distribution of GO enrichment annotation of target genes for YGS against PD-DT. The top biological process (BP) terms, cellular component (CC) terms, and molecular functions (MF) terms were exhibited according to the parameters of gene counts, p-value, and fold enrichment. The distinct shapes showed the difference in GO terms. The y-axis was the gene functional classification of GO, and the x-axis was the corresponding value of fold enrichment. There was a positive proportional relationship between the size and the counts of the target genes. Additionally, there was a correlation between the shade of color and the negative logarithm of the p-value, with brighter red shades indicating higher values.

**Fig. (7):**
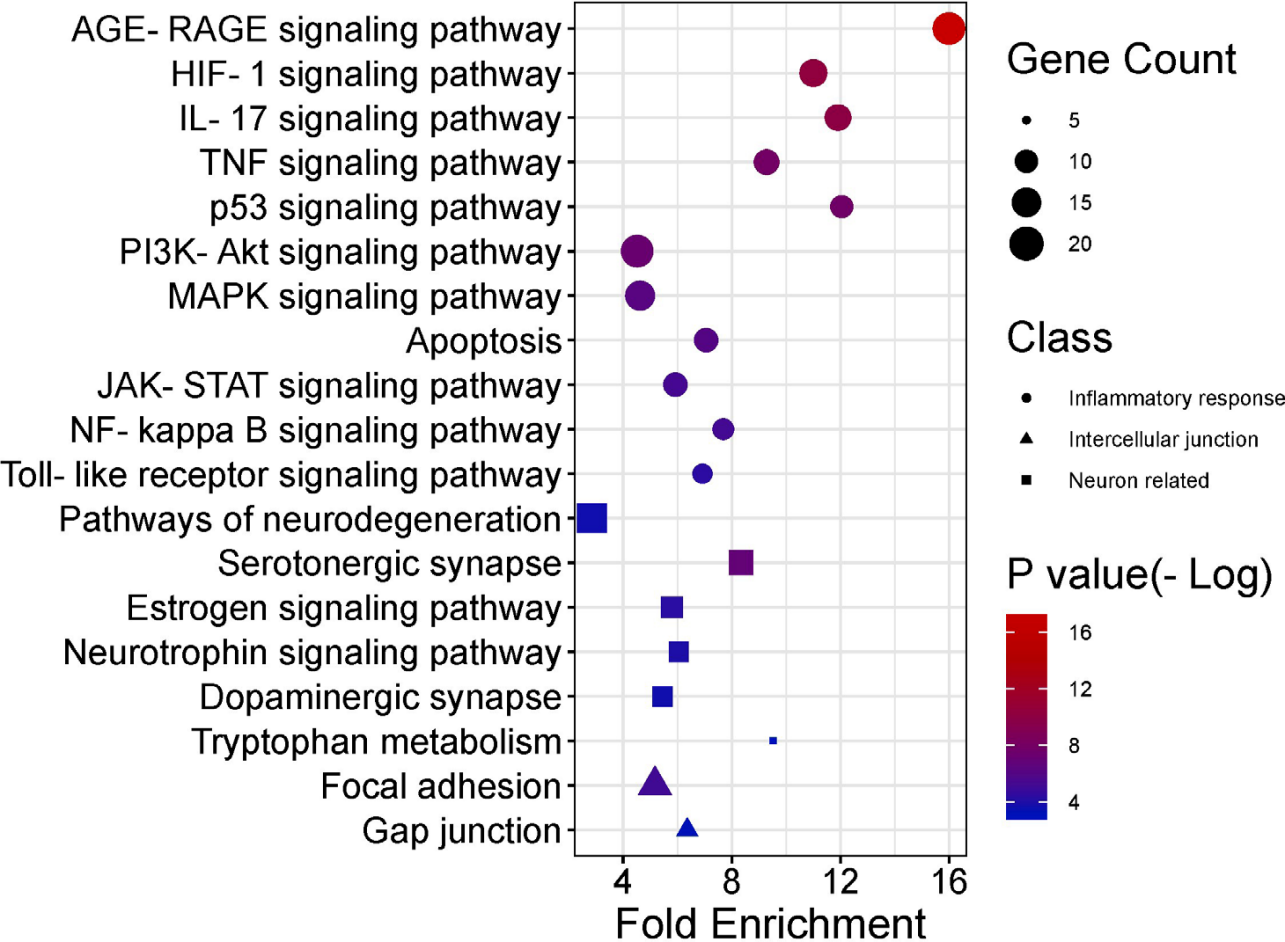
KEGG enrichment analysis of the target genes for YGS against PD-DT. The color scales indicate the different thresholds of adjusted *p*-values, and the sizes of the dots represent the gene count of each term. The y-axis was the classification of the KEGG pathway, and the x-axis was the level of fold enrichment. Also, the distinct shapes showed the related functional differences of each pathway.

**Fig. (9):**
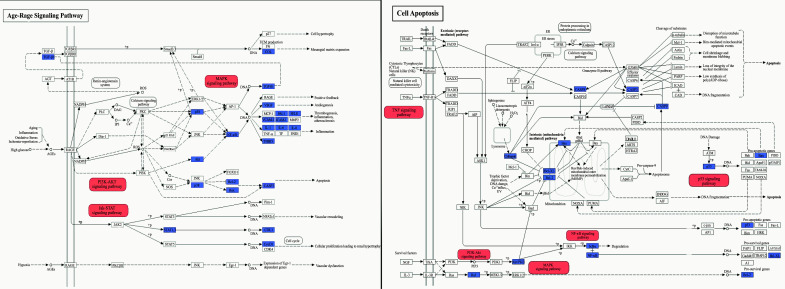
Potential target spots of YGS on regulating Age-Rage signaling and cell-apoptosis relevant pathways. Arrows indicate activation effects, and T-arrows indicate inhibition effects. The spots in purple represent targets regulated by YGS, and these spots serve as the essential regulatory factors for the red-highlighted pathways.

**Fig. (10):**
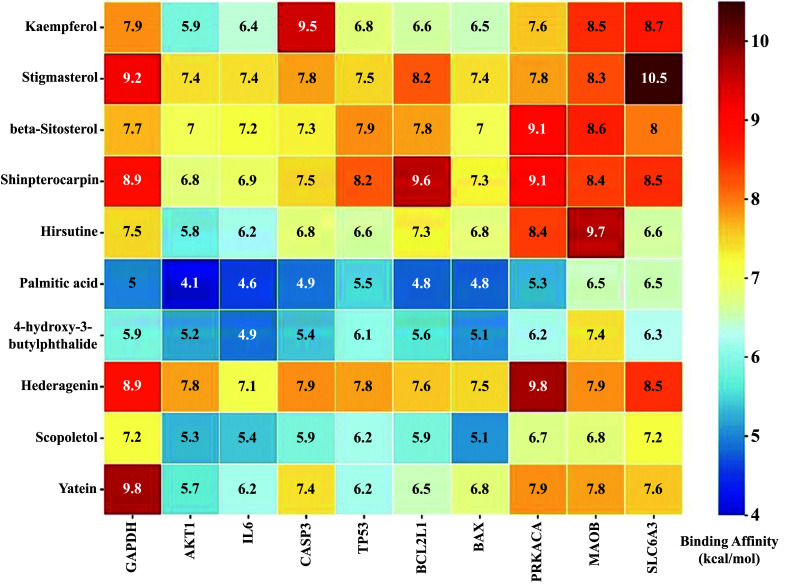
The heat map information of the compounds-targets binding affinity (showed in absolute value).

**Table 1:** The pre-docking preparation parameters of target proteins.

**Sample**	**PDB ID**	**Method**	**Resolution**	**R-free Value**
GAPDH	6YND	X-ray Diffraction	1.52 Å	0.2
AKT1	1UNQ	X-ray Diffraction	0.98 Å	0.179
IL6	7NXZ	X-ray Diffraction	2.0 Å	0.25
CASP3	4JJE	X-ray Diffraction	1.48 Å	0.174
TP53	6GGF	X-ray Diffraction	1.32 Å	0.148
BCL2L1	7LH7	X-ray Diffraction	1.4 Å	0.199
BAX	5W60	X-ray Diffraction	1.8 Å	0.238
PRKACA	7V0G	X-ray Diffraction	1.6 Å	0.163
MAOB	6FW0	X-ray Diffraction	1.6 Å	0.19
SLC6A3	6M0Z	X-ray Diffraction	2.88 Å	0.243

**Table 2:** The corresponding pharmacokinetic information and potential target genes of active compounds in YGS.

**S.No.**	**Compound Name**	**OB**	**DL**	**HL**	**Scientific Name**	**Related Target Gene**
G1	Isopteropodin	105.2	0.697	10.4	*Uncaria rhynchophylla*	ACHE,AR,ESR1,HSP90AA1,OPRM1,NOS2, KCNH2,PTGS1,PTGS2,SCN5A,PRSS1
G2	(2S,12Br)-Methyl 2-((E)-1-Oxobut -2-En-2-Yl)-1,2,6,7,12,12B- Hexahydroindolo[2,3-A]Quinolizine -3-arboxylate	42	0.603	12.3	*Uncaria rhynchophylla*	ACHE,DRD1,AR,HSP90AA1,OPRM1,NOS2, PPARG,KCNH2,PTGS1,PTGS2,SCN5A,PRSS1
G3	Yatein	51.7	0.649	8.88	*Uncaria rhynchophylla*	PDE10A,HSP90AA1,MET,PTPN22,KCNH2, PTGS1,PTGS2,SCN5A,KDR
G4	Corynoxine B	54.4	0.565	13.6	*Uncaria rhynchophylla*	HTR2A,ACHE,AR,HSP90AA1,OPRM1,PTGS1, PTGS2,SCN5A
G5	Corynoxine	57.8	0.565	13.1	*Uncaria rhynchophylla*	AR,HSP90AA1,KCNH2,PTGS1,PTGS2,SCN5A
G6	Angustidine	51.8	0.656	7.4	*Uncaria rhynchophylla*	HTR3A,ACHE,AR,HSP90AA1,NOS2,NOS2, KCNH2,PTGS1,PTGS2,PRSS1
G7	Corynoxeine	57.1	0.569	14.1	*Uncaria rhynchophylla*	HTR2A,ACHE,AR,HSP90AA1,KCNH2,PTGS1, PTGS2,SCN5A
G8	Delta-Hirsutine	41.6	0.642	13.2	*Uncaria rhynchophylla*	HTR2A,HTR2C,HTR3A,ACHE,AR,ADRB2, HSP90AA1,OPRM1,NOS2,PPARG,KCNH2, PTGS1,PTGS2,SCN5A
G9	Geissoschizinc Acid	49.9	0.597	11.1	*Uncaria rhynchophylla*	AR,ADRB2,HSP90AA1,NOS2,PPARG,KCNH2, PTGS1,PTGS2,SCN5A,PRSS1
G10	Hirsutaside A	70.3	0.806	16.4	*Uncaria rhynchophylla*	AR,HSP90AA1,PPARG,KCNH2,PTGS2, SCN5A,PRSS1
G11	Hirsutaside B	40.2	0.804	15.5	*Uncaria rhynchophylla*	HSP90AA1,MET,KCNH2,PTGS1,PTGS2,KDR
G12	Isocorynantheic Acid	72.3	0.597	12.8	*Uncaria rhynchophylla*	ACHE,AR,ADRB2,HSP90AA1,OPRM1,NOS2, PPARG,KCNH2,PTGS1,PTGS2,SCN5A
G13	Isorhyncophylline	47.3	0.565	12.6	*Uncaria rhynchophylla*	ACHE,AR,HSP90AA1,OPRM1,KCNH2,PTGS1, PTGS2,SCN5A
G14	Corynantheine	56.8	0.641	11.8	*Uncaria rhynchophylla*	HTR2C,HTR3A,ACHE,AR,ADRB2,ESR1, HSP90AA1,OPRM1,NOS2,NOS2,PPARG, KCNH2,PTGS1,PTGS2,SCN5A,PRSS1
G15	Rhynchophylline	41.8	0.565	13.2	*Uncaria rhynchophylla*	ACHE,AR,HSP90AA1,OPRM1,PTGS1, PTGS2,SCN5A
G16	Rhynchophylline A	68.6	0.69	9.97	*Uncaria rhynchophylla*	ACHE,AR,ESR1,HSP90AA1,OPRM1,NOS2, KCNH2,PTGS1,PTGS2,SCN5A
G17	Isopteropodine	78.3	0.748	11.7	*Uncaria rhynchophylla*	ACHE,AR,ESR1,HSP90AA1,OPRM1,NOS2, PTGS1,PTGS2,SCN5A
G18	Pteropodine	56.7	0.748	10.7	*Uncaria rhynchophylla*	ACHE,AR,ESR1,HSP90AA1,OPRM1, NOS2,PTGS2, SCN5A
G19	Vincoside Lactam	50.8	0.816	22.9	*Uncaria rhynchophylla*	AR,ADRB2,ESR1,HSP90AA1,NOS2, PPARG,KCNH2,PTGS2,SCN5A
G20	Yohimbine	46.4	0.81	9.36	*Uncaria rhynchophylla*	HTR2A,HTR2C,ACHE,AR,ADRB2, HSP90AA1,OPRM1, PPARG,KCNH2,PTGS1,PTGS2,SCN5A
D1	Acoradiene	36.7	0.429	8.41	*Angelicae sinensis Radix*	PTGS1,PTGS2,SLC6A3
D2	Beta-Chamigrene	31.9	0.38	8.59	*Angelicae sinensis Radix*	MAOB,PTGS1,PTGS2
D3	Beta-Sitosterol	36.9	0.751	5.35	*Angelicae sinensis Radix*	HTR2A,BAX,BCL2,ADRB2,CASP3,CASP8, CASP9,DRD1,HSP90AA1,MAP2,PRKACA, OPRM1,KCNH2,PTGS1,PTGS2,PON1,SCN5A, SLC6A4,TGFB1
D4	Beta-Terpinene	42.2	0.322	11.2	*Angelicae sinensis Radix*	ADH1C,PTGS1,PTGS2
D5	Isoeugenol	70.1	0.336	6.48	*Angelicae sinensis Radix*	MAOA,MAOB,ADRB2,IL2,NOS2,PTGS1,PTGS2, SLC6A3,RELA
D6	Alpha-acoradiene	40.9	0.372	9.04	*Angelicae sinensis Radix*	DRD1,PTGS1,PTGS2,SLC6A3
DF1	Palmitic Acid	33.6	0.81	9.26	*Angelicae sinensis Radix/* *Wolfiporia extensa*	ADH1C,BCL2,CTSD,PCYT1A,COL1A1,IL10, PTEN,PTGS1,PTGS2,RHO,SLC22A5
F1	16alpha-Hydroxydehydro trametenolic acid	30.9	0.812	6.81	*Wolfiporia extensa*	NR3C2
F2	2-Lauroleic Acid	31.4	0.044	5.68	*Wolfiporia extensa*	PTGS1
F3	Cerevisterol	37.9	0.77	5.31	*Wolfiporia extensa*	NR3C2
F4	Hederagenin	36.9	0.75	5.34	*Wolfiporia extensa*	PTGS1,SCN5A,PTGS2,PTGS1,SCN5A,PTGS2
F5	Trametenolic Acid	38.7	0.801	7.77	*Wolfiporia extensa*	NR3C2
CD1	Stigmasterol	43.8	0.756	5.57	*Radix bupleuri/* *Angelicae Sinensis Radix*	HTR2A,ADH1C,MAOA,MAOB,ADRB2,NR3C2, PTGS1,PTGS2,SCN5A,SLC6A3,PLAU,ALOX5, SLC25A13,GLUL,GCH1,GAMT,LDHB,REN,TPI1
C1	(+)-Anomalin	46	0.656	10	*Radix bupleuri*	BCHE,PTGS1,PTGS2
C2	3',4',5',3,5,6,7-Heptamethoxyflavone	31.9	0.593	15.5	*Radix bupleuri*	DRD1,PTGS1,PTGS2,SCN5A
C3	Areapillin	48.9	0.413	16.5	*Radix bupleuri*	ADH1C,ACHE,AR,ESR1,ESR2,PTGS2,F2,PRSS1, BCHE,ADH1C
C4	Cubebin	57.1	0.639	12.4	*Radix bupleuri*	MAOB,SLC6A3,NOS2,PTGS1,F2
C5	Isorhamnetin	49.6	0.306	14.3	*Radix bupleuri*	ACHE,MAOB,AR,BAX,BCL2,BCL2L1,ADRB2, EGFR,CCND1,HSP90AA1,PPARG,KCNH2,PTGS1, PTGS2,AKT1,SCN5A,MMP3,F2,RELA,PRSS1, VEGFA,MAOA
C6	Kaempferol	41.8	0.24	14.7	*Radix bupleuri*	ALOX5,CASP3,CASP8,CAV1,TP53,COL1A1, CDKN2A,CYP1A1,CYP1A2,CYP1B1,CYP3A4, SELE,COL1A1,HSPB1,HMOX1,HIF1A,ICAM1, IFNG,IL1A,IL1B,IL2,IL6,CXCL8,MMP1,MYC, NFKBIA,PPARG,SERPINE1,RAF1,ERBB2, RB1,STAT1,THBD,TGFB1,VCAM1,XDH
C7	Linoleyl Acetate	42.1	0.198	7.47	*Radix bupleuri*	ACHE,PTGS2
C8	Quercetin	46.4	0.275	14.4	*Radix bupleuri*	APRT,F13A1,EPHX1,BCHE,ALDOA,G6PD, GLUD1,GSTM1,GAPDH,HMOX1,HARS1, LDHB,MTR,NEK9,NDUFV2,PYCR1,TPI1, WARS2
C9	Spinasterol	42.9	0.756	6.46	*Radix bupleuri*	GBA2,PTGS2
B1	14-Acetyl-12-Senecioyl-2E,8Z,10E-Atractylentriol	63.3	0.299	6.42	*Atractylodis macrocephalae* *rhizoma*	PTGS2
B2	Beta-acetoxyatractylone	54	0.219	10.3	*Atractylodis macrocephalae* *rhizoma*	ACHE,AR,ADRB2,OPRM1,PTGS2,SCN5A
B3	Beta-ethoxy atractylenolide III	35.9	0.21	8.34	*Atractylodis macrocephalae* *rhizoma*	PTGS2
B4	Atractylenolide	68.1	0.271	7.16	*Atractylodis macrocephalae* *rhizoma*	IL1B,IL6,VEGFA
B5	Beta-Caryophyllene	39.7	0.289	11.7	*Atractylodis macrocephalae* *rhizoma*	IL6,PTGS1,PTGS2,SLC6A3
B6	Beta-Selinene	34.3	0.281	6.81	*Atractylodis macrocephalae* *rhizoma*	PTGS1,PTGS2
B7	Scopoletol	37.7	0.216	7.6	*Atractylodis macrocephalae* *rhizoma*	MAOB,ADRB2,PRKACA,NQO2,PTGS1,PTGS2
B8	Longipinene	53.2	0.324	12.5	*Atractylodis macrocephalae* *rhizoma*	PTGS2
X1	(L)-Alpha-Terpineol	48.7	0.307	11.3	*Chuanxiong rhizoma*	PTGS1,PTGS2,SLC6A3,HSP90AA1,SCN5A, SCN5A
X2	(R)-Linalool	41.8	0.226	6.47	*Chuanxiong rhizoma*	PTGS1,PTGS2,KDR,HSP90AA1,KCNH2,SCN5A
X3	4,7-Dihydroxy-3-Butylphthalide	106	0.196	4.63	*Chuanxiong rhizoma*	ADRB2,PTGS1,PTGS2,SLC6A3,SLC6A4,MAOB, HSP90AA1,PRKACA
X4	4-Hydroxy-3-Butylphthalide	70.3	0.28	4.8	*Chuanxiong rhizoma*	ADRB2,PTGS1,PTGS2,SLC6A3,SLC6A4,HTR2A, MAOB,DRD2,HSP90AA1,PRKACA,SCN5A
X5	Alpha-Selinene	41.8	0.199	9.15	*Chuanxiong rhizoma*	PTGS2
X6	Beta-Asarone	45.6	0.26	4.46	*Chuanxiong rhizoma*	ADRB2,PTGS1,PTGS2,SLC6A3,SLC6A4,HTR2A, DRD1,KCNH2,SCN5A
X7	Cerulignol	62.4	0.235	4.66	*Chuanxiong rhizoma*	ACHE,MAOA,ADRB2,PTGS1,PTGS2,SLC6A3, MAOB,DRD1,NOS2
X8	L-Bornyl Acetate	65.5	0.275	6.93	*Chuanxiong rhizoma*	PTGS2,DRD1
X9	Mandenol	41.9	0.193	5.38	*Chuanxiong rhizoma*	PTGS1,PTGS2
X10	Methyl Linoleate	41.9	0.167	6.05	*Chuanxiong rhizoma*	PTGS1,PTGS2
X11	Myricanone	40.5	0.512	4.38	*Chuanxiong rhizoma*	AR,ADRB2,ESR1,ESR2,GSK3B,MAPK14,PTGS1, PTGS2,KDR,HSP90AA1,NOS2, PPARG,KCNH2,SCN5A
X12	Perlolyrine	65.9	0.274	12.6	*Chuanxiong rhizoma*	PTGS2,NOS2
X13	Sitogluside	40.6	0.624	10.9	*Chuanxiong rhizoma*	HTR3A,ADRB2,PTGS1,PTGS2,HSP90AA1, KCNH2,SCN5A
X14	Wallichilide	42.3	0.706	10.9	*Chuanxiong rhizoma*	NR3C2,PTGS2
L1	(2S)-6-(2,4-Dihydroxyphenyl)- 2-(2-Hydroxypropan-2-Yl)- 4-Methoxy-2,3-Dihydrofuro[3,2-G]Chromen-7-One	60.2	0.634	4.31	*Glycyrrhizae radix et* *Rhizoma*	NOS2,F2,ESR1,AR,PPARG,PTGS2,KDR,ACHE, ESR2,MAPK14,GSK3B,PRSS1
L2	1-Methoxyphaseollidin	69.9	0.637	9.52	*Glycyrrhizae radix et* *Rhizoma*	NOS2,PTGS1,F2,KCNH2,ESR1,AR,SCN5A, PPARG,PTGS2,KDR,ADRB2,ESR2, MAPK14,GSK3B,HSP90AA1,PRSS1
L3	Gancaonin H	50.1	0.784	16.6	*Glycyrrhizae radix et* *Rhizoma*	ESR1,AR,PTGS2,KDR,HSP90AA1,PRSS1
L4	Glyasperins M	72.6	0.592	15.6	*Glycyrrhizae radix et* *Rhizoma*	NOS2,PTGS1,KCNH2,ESR1,AR,SCN5A, PPARG,PTGS2,KDR,ACHE,ESR2, GSK3B,HSP90AA1,PRKACA,PRSS1
L5	Glycyrol	90.7	0.668	9.84	*Glycyrrhizae radix et* *Rhizoma*	NOS2,ESR1,PPARG,PTGS2,KDR,MAPK14, GSK3B,F2
L6	Licoagrocarpin	58.8	0.584	9.44	*Glycyrrhizae radix et* *Rhizoma*	NOS2,PTGS1,F2,KCNH2,ESR1,AR,SCN5A, PPARG,PTGS2,ACHE,ADRB2,ESR2, MAPK14,GSK3B,HSP90AA1,PRSS1
L7	Licopyranocoumarin	80.3	0.653	8.45	*Glycyrrhizae radix et* *Rhizoma*	NOS2,F2,ESR1,AR,PPARG,PTGS2,KDR, ACHE,PRSS1
L8	Liquiritin	65.6	0.738	18	*Glycyrrhizae radix et* *Rhizoma*	PTGS2,KDR
L9	Phaseol	78.7	0.578	9.64	*Glycyrrhizae radix et* *Rhizoma*	F2,ESR1,AR,PPARG,PTGS2,KDR,MAPK14, GSK3B,HSP90AA1,PRKACA
L10	Shinpterocarpin	80.2	0.727	6.5	*Glycyrrhizae radix et* *Rhizoma*	NOS2,PTGS1,KCNH2,ESR1,AR,SCN5A, PPARG,PTGS2,HTR3A,ADRB2,OPRM1, ESR2,MAPK14,GSK3B,PRKACA,PRSS1
L11	Xambioona	54.8	0.874	14.5	*Glycyrrhizae radix et* *Rhizoma*	NOS2,ESR1,PTGS2,ESR2

**Table 3:** Topological analysis of the core active compounds in the components-compounds-targets network. (Sorted on Betweenness Centrality).

**NO.**	**Degree**	**Betweenness Centrality**	**Name**
CD1	21	0.0268	Stigmasterol
C6	37	0.0262	Kaempferol
D3	20	0.0188	Beta-sitosterol
L10	17	0.0161	Shinpterocarpin
X4	12	0.0144	4-Hydroxy-3-butylphthalide
DF1	13	0.0135	Palmitic acid
F4	7	0.0132	Hederagenin
G3	10	0.0128	Yatein
B7	7	0.0123	Scopoletol
G8	15	0.0122	Hirsutine

**Table 4:** Topological analysis of the core target protein in the PPI network.

**Name**	**Degree**	**Betweenness Centrality**
TP53	58	0.5232
SLC6A3	13	0.154
GAPDH	67	0.0834
MAOB	14	0.0593
AKT1	64	0.0567
BAX	19	0.0558
IL6	64	0.0515
BCL2L1	41	0.0435
PRKACA	18	0.0424
CASP3	58	0.0422

**Table 5:** Topological analysis of the GO functional enrichment analysis.

**Class**	**Description**	**Fold Enrichment**	***P* value (-log)**	**count**
BP	Positive Regulation of Transcription from RNA Polymerase II Promoter	3.8	8	26
BP	Response to Lipopolysaccharide	18	13.95	16
BP	Negative Regulation of Cell Proliferation	6.1	7.32	16
BP	Apoptotic Process	4.3	5.07	15
BP	Angiogenesis	9	7.69	13
BP	Cell Aging	9.9	6.82	11
BP	Inflammatory Response	4.7	3.95	11
CC	Cytoplasm	1.7	4.29	35
CC	Mitochondrion	3.4	6.95	26
CC	Neuron Projection	4.4	3.01	9
CC	Mitochondrial Outer Membrane	7.8	4.13	8
CC	Integral Component of Postsynaptic Membrane	25.5	6.53	7
CC	Integral Component of Presynaptic Membrane	19.7	5.85	7
CC	Focal Adhesion	3.1	1.58	7
MF	Protein Kinase Binding	5.7	7.4	17
MF	Ubiquitin Protein Ligase Binding	8.4	8.58	15
MF	Transcription Factor Binding	8.2	5.45	10
MF	Oxidoreductase Activity	7.1	4.95	10
MF	Cytokine Activity	7.1	3.88	8
MF	Protein Phosphatase Binding	12.9	4.76	7

**Table 6:** Topological analysis of pathways in KEGG enrichment analysis.

**Class**	**Description**	**Fold Enrichment**	***P* value (-log)**	**Count**
Inflammatory response	AGE-RAGE signaling pathway	15.99	17.24	20
Inflammatory response	HIF-1 signaling pathway	11	10.31	15
Inflammatory response	IL-17 signaling pathway	11.9	10.01	14
Inflammatory response	TNF signaling pathway	9.2	7.97	13
Inflammatory response	p53 signaling pathway	12.04	7.75	11
Inflammatory response	PI3K-Akt signaling pathway	4.51	7.3	20
Inflammatory response	MAPK signaling pathway	4.62	6.26	17
Inflammatory response	Apoptosis	7.05	6.08	12
Inflammatory response	JAK-STAT signaling pathway	5.92	5.33	12
Inflammatory response	NF-kappa B signaling pathway	7.68	5.3	10
Inflammatory response	Toll-like receptor signaling pathway	6.91	4.39	9
Neural function	Pathways of neurodegeneration	2.85	3.64	17
Neural function	Serotonergic synapse	8.34	6.82	12
Neural function	Estrogen signaling pathway	5.79	4.31	10
Neural function	Neurotrophin signaling pathway	6.04	3.97	9
Neural function	Dopaminergic synapse	5.45	3.66	9
Neural function	Tryptophan metabolism	9.51	2.76	5
Intercellular junction	Focal adhesion	5.17	5.2	13
Intercellular junction	Gap junction	6.36	3.12	7

## Data Availability

The data used to support the findings of this study are included in the article.

## LIST OF ABBREVIATIONS

YGS: Yi-Gai-San
PD-DT: Tremor-dominant Subtype of Parkinson's Disease
PPI: Protein-protein Interaction
BC: Betweenness Centrality
BP: Biological Process
CC: Cellular Component
CTD: Comparative Toxicogenomic Database
CCT: Components-Compounds-Targets
DC: Degree Centrality
DRD1: Dopamine Receptor D1
DRD2: Dopamine Receptor D2
GO: Gene Ontology
IL-6: Interleukin-6
KEGG: Kyoto Encyclopedia of Genes and Genomes
MAOB: Monoamine Oxidase Type B
TP53: Tumor Protein p53
SLC6A3: Solute Carrier Family 6 A3
DAT: Dopamine Transporter
GAPDH: Glyceraldehyde-3-phosphate Dehydrogenase
AKT: Serine/Threonine Kinase
BAX: BCL2 Associated X
IL6: Interleukin-6
BCL2: B-cell Lymphoma 2
PKA: Protein Kinase A
CASP3: Caspase-3
PPI: Protein-protein Interaction
SLC6A3: Solute Carrier Family 6 Member 3
DAVID: The Database for Annotation, Visualization, and Integrated Discovery
TTD: Therapeutic Target Database
TCM: Traditional Chinese Medicine
TCMSP: Traditional Chinese Medicine System Pharmacology

## References

[1] Bloem B.R., Okun M.S., Klein C. (2021). Parkinson’s disease.. Lancet.

[2] Hallett M. (2012). Parkinson’s disease tremor: Pathophysiology.. Parkinsonism Relat. Disord..

[3] Thenganatt M.A., Jankovic J. (2014). Parkinson disease subtypes.. JAMA Neurol..

[4] Xiong W., Li L.F., Huang L., Liu Y., Xia Z.C., Zhou X.X., Tang B.S., Guo J.F., Lei L.F. (2020). Different iron deposition patterns in akinetic/rigid-dominant and tremor-dominant Parkinson’s disease.. Clin. Neurol. Neurosurg..

[5] Jankovic J. (2018). Parkinson’s disease tremors and serotonin.. Brain.

[6] Bandres-Ciga S., Diez-Fairen M., Kim J.J., Singleton A.B. (2020). Genetics of Parkinson’s disease: An introspection of its journey towards precision medicine.. Neurobiol. Dis..

[7] Langston R.G., Beilina A., Reed X., Kaganovich A., Singleton A.B., Blauwendraat C., Gibbs J.R., Cookson M.R. (2022). Association of a common genetic variant with Parkinson’s disease is mediated by microglia.. Sci. Transl. Med..

[8] Armstrong M.J., Okun M.S. (2020). Diagnosis and treatment of parkinson disease.. JAMA.

[9] Stibe C.M.H., Kempster P.A., Lees A.J., Stern G.M. (1988). Subcutaneous apomorphine in parkinsonian on-off oscillations.. Lancet.

[10] Borovac J.A. (2016). Side effects of a dopamine agonist therapy for Parkinson’s disease: A mini-review of clinical pharmacology.. Yale J. Biol. Med..

[11] Li X., Zhang Y., Wang Y., Xu J., Xin P., Meng Y., Wang Q., Kuang H. (2017). The mechanisms of traditional Chinese medicine underlying the prevention and treatment of Parkinson’s disease.. Front. Pharmacol..

[12] Zhang G., Xiong N., Zhang Z., Liu L., Huang J., Yang J., Wu J., Lin Z., Wang T. (2015). Effectiveness of traditional Chinese medicine as an adjunct therapy for Parkinson’s disease: A systematic review and meta-analysis.. PLoS One.

[13] Zhang J., Ma Y., Shen X. (2013). Evaluation on the efficacy and safety of chinese herbal medication xifeng dingchan pill in treating Parkinson’s disease: Study protocol of a multicenter, open-label, randomized active-controlled trial.. J. Integr. Med..

[14] (2022). Taiwan Herbal Pharmacopeia.

[15] Shinno H., Utani E., Okazaki S., Kawamukai T., Yasuda H., Inagaki T., Inami Y., Horiguchi J. (2007). Successful treatment with Yi-Gan San for psychosis and sleep disturbance in a patient with dementia with Lewy bodies.. Prog. Neuropsychopharmacol. Biol. Psychiatry.

[16] Miyaoka T., Furuya M., Yasuda H., Hayashida M., Nishida A., Inagaki T., Horiguchi J. (2008). Yi-gan san for the treatment of neuroleptic-induced tardive dyskinesia: An open-label study.. Prog. Neuropsychopharmacol. Biol. Psychiatry.

[17] Hu S., Mak S., Zuo X., Li H., Wang Y., Han Y. (2018). Neuroprotection against MPP^+^-induced cytotoxicity through the activation of PI3-K/Akt/GSK3β/MEF2D signaling pathway by rhynchophylline, the major tetracyclic oxindole alkaloid isolated from **Uncaria rhynchophylla*.*. Front. Pharmacol..

[18] Zheng M., Chen M., Liu C., Fan Y., Shi D. (2021). Alkaloids extracted from *Uncaria rhynchophylla* demonstrate neuroprotective effects in MPTP-induced experimental parkinsonism by regulating the PI3K/Akt/mTOR signaling pathway.. J. Ethnopharmacol..

[19] Hatano T. (2014). An exploratory study of the efficacy and safety of yokukansan for neuropsychiatric symptoms in patients with Parkinson’s disease.. J. Neural. Transm.

[20] Jin C., Cho K.H., Kwon S., Lee H.G., Kim T.H., Jung W.S., Moon S.K., Cho S.Y., Kang B.K., Park J.M., Park H.J., Ko C.N. (2022). Effectiveness and safety of herbal medicine Ukgansan for clinical symptoms in Parkinson’s disease: A pilot, randomized, assessor-blinded clinical trial.. Front. Neurol..

[21] Miyaoka T., Furuya M., Horiguchi J., Wake R., Hashioka S., Tohyama M., Mori N., Minabe Y., Iyo M., Ueno S., Ezoe S., Murotani K., Hoshino S., Seno H. (2015). Efficacy and safety of yokukansan in treatment-resistant schizophrenia: A randomized, double-blind, placebo-controlled trial (a positive and negative syndrome scale, five-factor analysis).. Psychopharmacology.

[22] Chi Z., Guo R-J., Ren F-F. (2022). Network pharmacological analysis on the active ingredients of Yigan Powder in treating Alzheimer’s disease with depressive disorder.. Hainan Yixueyuan Xuebao.

[23] Yang H., Zhang W., Huang C., Zhou W., Yao Y., Wang Z., Li Y., Xiao W., Wang Y. (2014). A novel systems pharmacology model for herbal medicine injection: A case using reduning injection.. BMC Complement. Altern. Med..

[24] Zhang Y., Yuan T., Li Y., Wu N., Dai X. (2021). Network pharmacology analysis of the mechanisms of compound herba sarcandrae (*Fufang Zhongjiefeng*) aerosol in chronic pharyngitis treatment.. Drug Des. Devel. Ther..

[25] Zhang R., Zhu X., Bai H., Ning K. (2019). Network pharmacology databases for traditional Chinese medicine: Review and assessment.. Front Pharmacol.

[26] Green O., Bader D.A. (2013). Faster betweenness centrality based on data structure experimentation.. Procedia Comput. Sci..

[27] Alighiarloo S.N., Taghizadeh M., Tavirani R.M., Goliaei B., Peyvandi A.A. (2014). Protein-protein interaction networks (PPI) and complex diseases.. Gastroenterol. Hepatol. Bed Bench.

[28] von Mering C. (2005). STRING: Known and predicted protein-protein associations, integrated and transferred across organisms.. Nucleic Acids Res..

[29] Harris M. A., J Clark, Ireland A, Lomax J., Ashburner M., Foulger R., Eilbeck K., Lewis S., Marshall B., Mungall C., Richter J., Rubin G.M., Blake J.A., Bult C., Dolan M., Drabkin H., Eppig J.T., Hill D.P., Ni L., Ringwald M., Balakrishnan R. (2004). The Gene Ontology (GO) database and informatics resource.. Nucleic Acids Res..

[30] Trott O., Olson A.J. (2010). AutoDock Vina: Improving the speed and accuracy of docking with a new scoring function, efficient optimization, and multithreading.. J. Comput. Chem..

[31] Kleywegt G.J., Jones A.T. (1997). Model building and refinement practice.. Methods in Enzymology..

[32] Read R.J. (2011). A new generation of crystallographic validation tools for the protein data bank.. Structure.

[33] Kushida H., Matsumoto T., Ikarashi Y. (2021). Properties, pharmacology, and pharmacokinetics of active indole and oxindole alkaloids in *Uncaria* hook.. Front. Pharmacol..

[34] Alvira D., Tajes M., Verdaguer E., Castroviejo A.D., Folch J., Camins A., Pallas M. (2006). Inhibition of the cdk5/p25 fragment formation may explain the antiapoptotic effects of melatonin in an experimental model of Parkinson’s disease.. J. Pineal Res..

[35] Haque M.N., Hannan M.A., Dash R., Choi S.M., Moon I.S. (2021). The potential LXRβ agonist stigmasterol protects against hypoxia/reoxygenation injury by modulating mitophagy in primary hippocampal neurons.. Phytomedicine.

[36] Mongkolpobsin K., Sillapachaiyaporn C., Nilkhet S., Tencomnao T., Baek S.J. (2023). Stigmasterol isolated from Azadirachta indica flowers attenuated glutamate-induced neurotoxicity *via* downregulation of the Cdk5/p35/p25 signaling pathway in the HT-22 cells.. Phytomedicine.

[37] Pan X., Liu X., Zhao H., Wu B., Liu G. (2020). Antioxidant, anti-inflammatory and neuroprotective effect of kaempferol on rotenone-induced Parkinson’s disease model of rats and SH-S5Y5 cells by preventing loss of tyrosine hydroxylase.. J. Funct. Foods.

[38] Abdullah A., Ravanan P. (2018). Kaempferol mitigates endoplasmic reticulum stress induced cell death by targeting caspase 3/7.. Sci. Rep..

[39] Filomeni G., Graziani I., De Zio D., Dini L., Centonze D., Rotilio G., Ciriolo M.R. (2012). Neuroprotection of kaempferol by autophagy in models of rotenone-mediated acute toxicity: Possible implications for Parkinson’s disease.. Neurobiol. Aging.

[40] Wu A.G., Zeng W., Wong V.K.W., Zhu Y.Z., Lo A.C.Y., Liu L., Law B.Y.K. (2017). Hederagenin and α-hederin promote degradation of proteins in neurodegenerative diseases and improve motor deficits in MPTP-mice.. Pharmacol. Res..

[41] Karthikkeyan G., Pervaje R., Pervaje S.K., Prasad T.S.K., Modi P.K. (2021). Prevention of MEK-ERK-1/2 hyper-activation underlines the neuroprotective effect of *Glycyrrhiza glabra* L. (Yashtimadhu) against rotenone-induced cellular and molecular aberrations.. J. Ethnopharmacol..

[42] Muchandi A.A., Dhawale S.C. (2018). Protective effects of ethanolic extract of *Piper cubeba* L. on D-galactose induced neuronal lipofuscinogenesis in albino rats.. Sci. Eng. Health Stud..

[43] Sanguanphun T., Promtang S., Sornkaew N., Niamnont N., Sobhon P., Meemon K. (2023). Anti-parkinson effects of *Holothuria leucospilota*-derived palmitic acid in *Caenorhabditis elegans* model of Parkinson’s disease.. Mar. Drugs.

[44] Tian Q., Wang L., Sun X., Zeng F., Pan Q., Xue M. (2019). Scopoletin exerts anticancer effects on human cervical cancer cell lines by triggering apoptosis, cell cycle arrest, inhibition of cell invasion and PI3K/AKT signalling pathway.. J. BUON Off. J. Balk. Union Oncol..

[45] Zhu S., Jiao W., Xu Y., Hou L., Li H., Shao J., Zhang X., Wang R., Kong D. (2021). Palmitic acid inhibits prostate cancer cell proliferation and metastasis by suppressing the PI3K/Akt pathway.. Life Sci..

[46] Jung H.Y., Nam K.N., Woo B.C., Kim K.P., Kim S.O., Lee E.H. (2013). Hirsutine, an indole alkaloid of *Uncaria rhynchophylla*, inhibits inflammation-mediated neurotoxicity and microglial activation.. Mol. Med. Rep..

[47] Zheng M., Chen M., Wang W., Zhou M., Liu C., Fan Y., Shi D. (2021). Protection by rhynchophylline against MPTP/MPP^+^-induced neurotoxicity *via* regulating PI3K/Akt pathway.. J. Ethnopharmacol..

[48] Terada K., Matsushima Y., Matsunaga K., Takata J., Karube Y., Ishige A., Chiba K. (2017). The Kampo medicine Yokukansan (YKS) enhances nerve growth factor (NGF)-induced neurite outgrowth in PC12 cells.. Bosn. J. Basic Med. Sci..

[49] Chen L., Huang Y., Yu X., Lu J., Jia W., Song J., Liu L., Wang Y., Huang Y., Xie J., Li M. (2021). Corynoxine protects dopaminergic neurons through inducing autophagy and diminishing neuroinflammation in rotenone-induced animal models of Parkinson’s disease.. Front. Pharmacol..

[50] Doo A.R., Kim S.N., Park J.Y., Cho K.H., Hong J., Eun-Kyung K., Moon S.K., Jung W.S., Lee H., Jung J.H., Park H.J. (2010). Neuroprotective effects of an herbal medicine, Yi-Gan San on MPP+/MPTP-induced cytotoxicity *in vitro* and *in vivo.*. J. Ethnopharmacol..

[51] Xian Y.F., Lin Z.X., Mao Q.Q., Ip S.P., Su Z.R., Lai X.P. (2012). Protective effect of isorhynchophylline against β-amyloid-induced neurotoxicity in PC12 cells.. Cell. Mol. Neurobiol..

[52] Zhao Y.R., Qu W., Liu W.Y., Hong H., Feng F., Chen H., Xie N. (2015). YGS40, an active fraction of Yi-Gan San, reduces hydrogen peroxide-induced apoptosis in PC12 cells.. Chin. J. Nat. Med..

[53] Beg T., Jyoti S., Naz F., Rahul, Ali F., Ali S.K., Reyad A.M., Siddique Y.H. (2018). Protective effect of kaempferol on the transgenic drosophila model of Alzheimer’s disease.. CNS Neurol. Disord. Drug Targets.

[54] Sekar S., Taghibiglou C. (2020). Nuclear accumulation of GAPDH, GluA2 and p53 in post-mortem substantia nigral region of patients with Parkinson’s disease.. Neurosci. Lett..

[55] Tatton N.A. (2000). Increased caspase 3 and Bax immunoreactivity accompany nuclear GAPDH translocation and neuronal apoptosis in Parkinson’s disease.. Exp. Neurol..

[56] Yamaguchi K., Yamazaki S., Kumakura S., Someya A., Iseki M., Inada E., Nagaoka I. (2020). Yokukansan, a Japanese herbal medicine, suppresses substance P-induced production of interleukin-6 and interleukin-8 by human U373 MG glioblastoma astrocytoma cells.. Endocr. Metab. Immune Disord. Drug Targets.

[57] Ebisawa S., Andoh T., Shimada Y., Kuraishi Y. (2015). Yokukansan improves mechanical allodynia through the regulation of interleukin-6 expression in the spinal cord in mice with neuropathic pain.. Evid. Based Complement. Alternat. Med..

[58] Lian T.H., Guo P., Zuo L.J., Hu Y., Yu S.Y., Yu Q.J., Jin Z., Wang R.D., Li L.X., Zhang W. (2019). Tremor-dominant in parkinson disease: The relevance to iron metabolism and inflammation.. Front. Neurosci..

[59] Derk J., MacLean M., Juranek J., Schmidt A.M. (2018). The receptor for advanced glycation endproducts (RAGE) and mediation of inflammatory neurodegeneration.. J. Alzheimers Dis. Parkinsonism.

[60] Tang X., Lu J., Chen H., Zhai L., Zhang Y., Lou H., Wang Y., Sun L., Song B. (2021). Underlying mechanism and active ingredients of tianma gouteng acting on cerebral infarction as determined *via* network pharmacology analysis combined with experimental validation.. Front. Pharmacol..

[61] Banerjee P., Eckert A.O., Schrey A.K., Preissner R. (2018). ProTox-II: A webserver for the prediction of toxicity of chemicals.. Nucleic Acids Res..

[62] Bickerton G.R., Paolini G.V., Besnard J., Muresan S., Hopkins A.L. (2012). Quantifying the chemical beauty of drugs.. Nat. Chem..

[63] Hou W.C., Lin R.D., Chen C.T., Lee M.H. (2005). Monoamine oxidase B (MAO-B) inhibition by active principles from *Uncaria rhynchophylla*.. J. Ethnopharmacol..

[64] Ishida Y., Ebihara K., Tabuchi M., Imamura S., Sekiguchi K., Mizoguchi K., Kase Y., Koganemaru G., Abe H., Ikarashi Y. (2016). Yokukansan, a traditional japanese medicine, enhances the L-DOPA-induced rotational response in 6-hydroxydopamine-lesioned rats: Possible inhibition of COMT.. Biol. Pharm. Bull..

[65] Xu Y., Wang R., Hou T., Li H., Han Y., Li Y., Xu L., Lu S., Liu L., Cheng J., Wang J., Xu Q., Liu Y., Liang X. (2023). Uncariphyllin A-J, indole alkaloids from *Uncaria rhynchophylla* as antagonists of dopamine D2 and Mu opioid receptors.. Bioorg. Chem..

[66] Zhong Y., Liu H., Liu G., Zhao L., Dai C., Liang Y., Du J., Zhou X., Mo L., Tan C., Tan X., Deng F., Liu X., Chen L. (2022). A review on pathology, mechanism, and therapy for cerebellum and tremor in Parkinson’s disease.. NPJ Parkinsons Dis..

[67] Ahsas Goyal W., Chisti W., Verma A., Agrawal N., Bansal K. (2023). The role of the serotonergic system of the brain in the pathogenesis of Parkinson’s disease.. Neurochem. J..

[68] Dirkx M.F., Bologna M. (2022). The pathophysiology of Parkinson’s disease tremor.. J. Neurol. Sci..

[69] Luo T., Lu Y., Yan S., Xiao X., Rong X., Guo J. (2020). Network pharmacology in research of Chinese medicine formula: Methodology, application and prospective.. Chin. J. Integr. Med..

[70] Wang X., Wang Z.Y., Zheng J.H., Li S. (2021). TCM network pharmacology: A new trend towards combining computational, experimental and clinical approaches.. Chin. J. Nat. Med..

